# Sub-annual bomb radiocarbon records from trees in northern Israel

**DOI:** 10.1038/s41598-023-46144-6

**Published:** 2023-11-01

**Authors:** Harsh Raj, Yael Ehrlich, Lior Regev, Eugenia Mintz, Elisabetta Boaretto

**Affiliations:** D-REAMS Radiocarbon Laboratory, Scientific Archaeology Unit, Weizmann Institute of Science, Rehovot, Israel

**Keywords:** Plant sciences, Biogeochemistry

## Abstract

Spatial and temporal variations in the atmospheric bomb radiocarbon make it a very useful tracer and a dating tool. With the introduction of more atmospheric bomb radiocarbon records, the spatial and temporal changes in bomb radiocarbon are becoming clearer. Bomb radiocarbon record from a pine tree in the northern Israel region shows that the Δ^14^C level in the region is closer to the northern hemisphere zone (NH) 1 as compared to the northern hemisphere zone (NH) 2. A comparison of this pine's Δ^14^C record with a nearby olive tree's Δ^14^C values also highlights changes in the growing season of the olive wood from one year to the other. The observation suggests that olive wood ^14^C ages can show offset compared to the IntCal curve, and thus they should be interpreted cautiously.

## Introduction

Radiocarbon, a radioactive isotope of carbon, is mainly produced in the earth’s atmosphere due to the interaction between neutrons associated with cosmic rays and nitrogen in the atmosphere^1^. Thermal neutrons were also produced anthropogenically during atmospheric nuclear weapon tests in 1950s and 1960s, which interacted with nitrogen to form radiocarbon^2,3^. Immediately after its production, radiocarbon (^14^C) oxidizes to^14^CO and ^14^CO_2_ subsequently^4,5^. The ^14^CO_2_ in the atmosphere enters different reservoirs like the ocean and biosphere through carbon cycle pathways. Initially, it was assumed that ^14^C in the atmosphere had been homogeneously distributed, but later, several studies reported spatial heterogeneity or offsets in atmospheric ^14^C values^6–10^.

The spatial heterogeneity in atmospheric ^14^C levels amplified when ^14^C concentration spiked during the 1950s and 1960s^11–16^. During this period, multiple atmospheric nuclear bomb tests were carried out, most of which were in the northern hemisphere^17–20^. It added large excess of ^14^C into the northern stratosphere, which was then transported to the northern troposphere and subsequently across the equator to the southern hemisphere ^21–24^. This addition almost doubled the ^14^C levels in the atmosphere^14,15,25^, which was observed in different atmospheric ^14^C measurements from both hemispheres during this period^24–29^. Due to stratospheric input of bomb-produced excess ^14^C in the northern hemisphere and its distribution by atmospheric circulations, a latitudinal gradient in atmospheric ^14^C level was created between the north and south hemispheres^,13,16,21^. 100–500 ‰ higher ^14^C levels were observed in the northern hemisphere compared to the southern hemisphere, and even within the northern hemisphere, decreasing trend was observed in ^14^C levels from high to low latitudes^12,16^. Large fluxes of excess ^14^C from the atmosphere to the ocean and biosphere during the bomb peak probably also contributed to regional atmospheric ^14^C variations^13^. Latitudinal gradients of ^14^C levels for a few years following the nuclear tests were dominantly influenced by stratospheric ^14^C input, and after the late 1960s, ocean and biosphere related ^14^C fluxes also contributed significantly to the latitudinal ^14^C gradient^16,30,31^. In recent years, fossil fuel emissions influenced the spatial distribution of atmospheric ^14^C level^31,32^.

In the past few decades, bomb ^14^C has been applied as a useful tracer to study atmospheric, terrestrial, and oceanic processes^13,20,33–39^. Bomb ^14^C also facilitated the dating of recent organic materials^40,41^. For the use of bomb ^14^C in the carbon cycle modelling and age calibration, Hua et al.^14^ defined different zones in the atmosphere based on the atmospheric ^14^C concentration between different latitudes. The boundary between these zones has been refined after the introduction of more radiocarbon records, and it led to a better understanding of radiocarbon distribution in the atmosphere^15^. Currently, there are three zones defined in the northern hemisphere, namely northern hemisphere (NH) zones 1, 2 and 3. The boundary between NH zone 1 and 2 is defined around 40°N by the Ferrel cell-Hedley cell boundary^14^, and the boundary between NH zone 2 and 3 is defined by TLPB (Tropical Low Pressure Belt) over continents^15^.

In spite of several measurements, there exist regions on the global map which are poorly represented by atmospheric ^14^C records during the bomb ^14^C period. To improve bomb ^14^C application as a tracer or dating tool, atmospheric ^14^C records from such underrepresented regions need to be studied. One such region is the eastern Mediterranean region in NH zone 2, which is represented by only about one year of atmospheric ^14^C measurement during the bomb ^14^C period^15^. The other nearest record in NH zone 2 is from the eastern coast of the Atlantic Ocean^15^. Therefore, more terrestrial bomb ^14^C records from this region are required to better understand the atmospheric bomb ^14^C distribution in the region. Tree ring cellulose-based ^14^C records can act as a reliable proxy for atmospheric ^14^C levels if the timing of ring growth (cellulose formation) is known and if stored photosynthate carbon is not used by tree for wood production. It becomes more relevant in case of high-resolution radiocarbon records*.* In present study, pine tree wood from northern Israel has been studied for its sub-annual ^14^C concentration around the bomb ^14^C peak period. These values have been compared with other atmospheric and terrestrial ^14^C records from the northern hemisphere to better understand the distribution of ^14^C in the atmosphere. Further, the analyzed pine ^14^C record is used to understand the growing season of olive wood from the same region.

## Results

### Growth period of earlywood and latewood

In this study, the reported pine tree (*Pinus halepensis,* HAN 5B) record from Havat Hanania (northern Israel) spans between the years 1964 and 1968. The annual rings in the pine wood were clearly visible (Figure S1). The wood sample showed distinct colour differences between earlywood and latewood, which helped in counting the growth year for each annual ring. Samples were sliced using a microtome, and about 4 sub-samples were obtained from each annual ring. A total of 20 contiguous samples were obtained and processed for alpha-cellulose extraction. These 20 alpha-cellulose samples were analyzed for radiocarbon (Δ^14^C) and stable carbon isotope (δ^13^C) measurements. Within each annual ring of *Pinus halepensis*, the growth season for earlywood and latewood samples was defined as per growth data of unwatered Pinus halepensis by Liphschitz et al.^42^, which shows two inactive or rest periods each year. Liphschitz et al.^42^ studied *Pinus halepensis* from Ilanoth, Israel and observed that in unwatered *Pinus halepensis* the earlywood grows during autumn and late winter, while its latewood grows during spring and early summer. They suggested that in winter the cambium activity of *Pinus halepensis* stops due to a drop in temperature and then again it stops in summer. As our sample location is a few kilometers away from Ilanoth and is not irrigated, we assume a similar growing season for the *Pinus halepensis* samples analyzed in this study. As local rainfall and temperature records do not show anomalously wet or dry years were observed between 1964 and 1968, we assumed the same wood growth season for each year. The earlywood samples were assigned to autumn and late winter to early spring period, and latewood samples were assigned to late spring and early summer period (Figure S2).

### Stable carbon isotope ratio in Havat Hanania pine

Results of stable carbon isotope measurements are reported in terms of δ^13^C, denoting deviations of stable carbon isotope ratio of sample from VPDB standard in per mil unit (‰). The δ^13^C values of analyzed samples vary between −25.2 ‰ and −20.2 ‰ (Table S1), with a mean value of −22.5 ± 1.4 ‰. A clear sub-annual cycle is observed in the pine δ^13^C values. Relatively higher δ^13^C values are observed in the latewood representing late spring to early summer, and relatively lower δ^13^C values are observed in the later part of earlywood, representing late winter to early spring (Figure S3). The timing of seasonal maxima and minima is similar to that of the seasonal variation in δ^13^C of atmospheric CO_2_ in the northern hemisphere^43^. However, the observed amplitude of the variation is much larger in the tree δ^13^C values, which is a result of fractionation during CO_2_ fixation by plants and is influenced by various environmental parameters^44,45^. It is observed that the amplitude and timing of Havat Hanania pine δ^13^C are comparable to the δ^13^C variations observed in *Pinus halepensis* from the Yatir forest in Israel^45^, wherein the lowest δ^13^C values occur in cooler periods and highest during warmer periods. The similarity in δ^13^C variation of analyzed pine and Yatir forest pines also supports the growth season assignment of pine wood based on Liphschitz et al.^42^.

### Radiocarbon in Havat Hanania Pine

Radiocarbon results are reported as Δ^14^C (‰), which are corrected for their age and isotopic fractionation based on δ^13^C values, following conventions of Stuiver and Polach^46^. The Δ^14^C values of Havat Hanania pine (HAN 5B) samples vary between 915 ‰ and 584 ‰ with evident sub-annual variations (Figure S2). These sub-annual variations are superimposed on a typical long-term post-bomb period Δ^14^C declining trend, which starts after the Δ^14^C peak in the spring and early summer of 1964. The peak value reaches 915 ± 3 ‰ during 1964, and after that, the Δ^14^C values show a decline rate of about 69 ‰yr^-1^.

**Figure 1:**
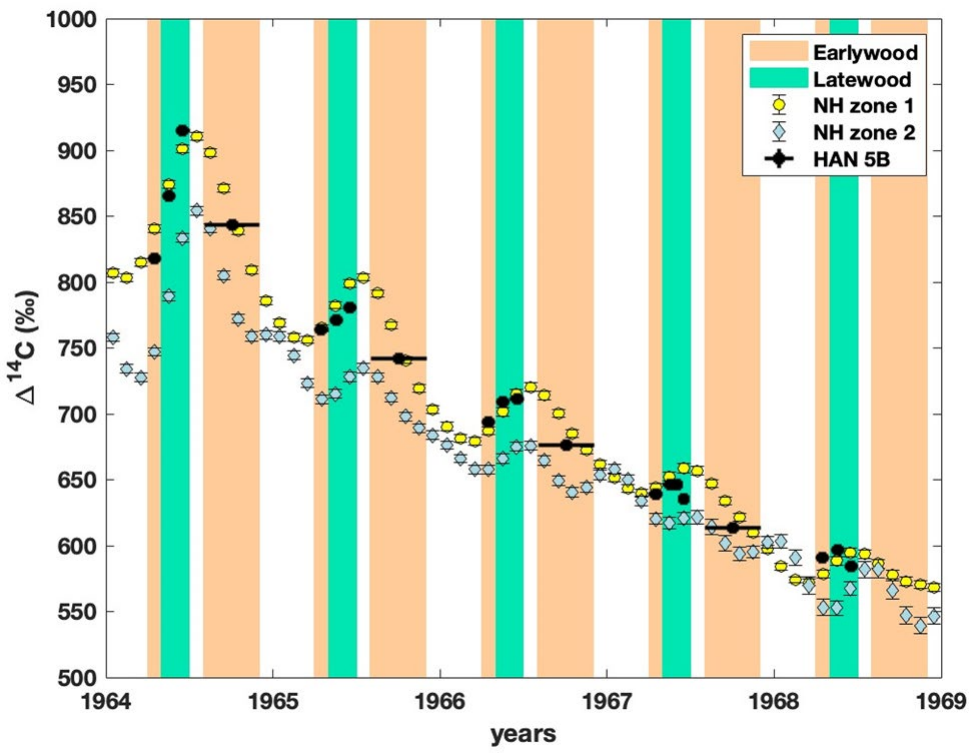
Δ^14^C values of Havat Hanania pine (HAN 5B) earlywood and latewood samples along with monthly Δ^14^C values from NH zone 1 and 2^15^ between the years 1964 and 1968. The time period of earlywood and latewood growth of HAN 5B (*Pinus halepensis*) is based on Liphschitz et al.^42^.

Based on the bomb Δ^14^C levels, Hua et al.^15^ grouped all atmospheric Δ^14^C records spatially into five different zones. The location of Havat Hanania (Israel) falls within NH zone 2^15^. Therefore, Δ^14^C records of HAN 5B are compared with monthly Δ^14^C data for NH zone 2 (Fig. 1). The comparison shows that between 1964 and 1968, HAN 5B Δ^14^C values mostly remained higher than monthly NH zone 2 Δ^14^C values. However, the HAN 5B Δ^14^C values match better with the monthly NH zone 1 Δ^14^C values. The good match between HAN 5B and NH zone 1 Δ^14^C values also implies that the growth season assignment of pine wood samples based on information from Liphschitz et al.^42^ is reasonable. The pine latewood Δ^14^C values are higher than adjacent earlywood Δ^14^C values for the analyzed period. These differences cannot arise due to the deposition of stored (older) carbon. The match between atmospheric and wood Δ^14^C values indicates that the difference between pine earlywood and latewood Δ^14^C values is related to atmospheric Δ^14^C changes. It is in conformity with the observations of Grootes et al.^47^ and Svarva et al.^48^ that coniferous trees can be good archives of sub-annual atmospheric ^14^C changes. Bomb ^14^C, majorly produced in the stratosphere, was transported to the troposphere mainly during the spring and summer^15^. This increased the atmospheric Δ^14^C values during spring and summer compared to the autumn and winter seasons. These sub-annual fluctuations are well recorded by the analyzed pine tree from the northern Israel.

## Discussion

The NH zone 1 consists of Δ^14^C records from mid to high latitude regions, and the NH zone 2 consists of Δ^14^C records between 45°N and TLPB (Tropical Low Pressure Belt) mean position during boreal summer^15^. In NH zone 1, atmospheric Δ^14^C records that span the years 1964 to 1968 are from Fruholmen, Norway^26^, Vermunt, Austria^27^ and China Lake, USA^49–53^. In NH zone 2, atmospheric Δ^14^C records from Izana, Mas Palomas and Santiago de Compostela in Spain and Dakar in Senegal^15,26^ span more than one year between 1964 and 1968. When these individual atmospheric records from NH zone 1 are compared with the Δ^14^C records of Havat Hanania (HAN 5B), it is observed that the HAN 5B record is in good agreement with them (Figure S4). Atmospheric records from NH zone 2 show more spread compared to NH zone 1. It is also observed that the HAN 5B values are higher than most of the individual atmospheric records from NH zone 2 (Figure S4). The difference of HAN 5B Δ^14^C values from contemporaneous monthly Δ^14^C values of each zone was calculated. The difference between HAN 5B and NH zone 1 Δ^14^C values ranges from −24 ‰ to 14 ‰ with a mean value of −4 ± 11 ‰. But the difference between HAN 5B and NH zone 2 Δ^14^C values was much higher, ranging between 14 ‰ to 82 ‰ with a mean value of 43 ± 20 ‰. This again suggests that the HAN 5B record is closer to NH zone 1 values.

To check for the goodness of fit between Δ^14^C values of HAN 5B and atmospheric curves (NH zones1 and 2) χ^2^ value was estimated. As per Ramsey et al.^54^, the χ^2^ value can be calculated using the following formula,

$$\upchi 2=\sum \frac{{({H}_{i}-{A}_{i})}^{2}}{({\sigma }_{{H}_{i}}^{2}+{\sigma }_{{A}_{i}}^{2})}$$

Here, H_i_ is the Δ^14^C values of HAN 5B, and A_i_ is the Δ^14^C values of the atmosphere curve (NH zone 1 or 2) for the contemporaneous period. σ represents the uncertainty associated with H_i_ and A_i_ values. The uncertainty of HAN 5B Δ^14^C record is between 2 and 3 ‰ and the uncertainty in the compiled monthly Δ^14^C values of NH zone 1 and 2 vary between 2 and 7 ‰^15^. Considering the uncertainty of 1σ in the atmospheric curves, χ^2^ values of 161.28 and 2389.24 were obtained for the atmospheric curve of NH zone 1 and 2, respectively. The reduced χ^2^ value in both cases is greater than 1. This suggests that within 1σ HAN 5B do not fit either of the NH 1 or 2 values significantly but Δ^14^C values of HAN 5B are closer to NH zone 1 Δ^14^C values compared to NH zone 2 Δ^14^C values. When 3σ uncertainty in the atmospheric curves was used, χ^2^ values of 17.92 and 265.47 were obtained for the atmospheric curve of NH zone 1 and 2, respectively. Now, the reduced χ^2^ value for the NH zone 1 curve is 0.90 (n = 20), and for the NH zone 2 curve, the reduced χ^2^ value is 13.7. These observations indicate that HAN 5B Δ^14^C values better fit NH zone 1 values^26,27,49–53^.

Between 1955 and 1967, due to the injection of bomb ^14^C into the troposphere, a gradient is observed from high to low latitudes^13,15^. The highest Δ^14^C values are observed in the mid to high-latitude region of the northern hemisphere and decrease southwards^15^. The bomb ^14^C distribution is influenced by atmospheric circulations, based on which Hua et al.^14^ defined different zones and boundaries between them. The NH zone 1 and 2 was separated by Ferrel cell–Hadley cell boundaries roughly around 40° N based on available Δ^14^C records^14^. With the inclusion of tree-ring Δ^14^C records from western Oregon, USA^55^ and Washington state, USA^47^, this boundary was modified. The current boundary between NH zone 1 and 2 over northwestern USA is considered to be between these two tree sites and south of China Lake (35° N) because China Lake Δ^14^C record is closer to NH zone 1 and higher than NH zone 2 Δ^14^C record mostly during winter-spring^14,15^. The present study location, Havat Hanania (32° N), is at about similar latitude as that of China Lake, and its atmospheric bomb Δ^14^C record is also closer to NH zone 1 values and relatively higher than NH zone 2 values. The monthly wind climatology map shows that the study location receives wind from higher latitudes in the north during summer (Figure S5). While during winter, the winds are from the west, mainly from the Mediterranean Sea. The winds during summer can bring ^14^C enriched CO_2_ from higher latitudes, explaining the good agreement between Havat Hanania and NH zone 1 atmospheric Δ^14^C record. These observations suggest that the present study area lies within NH zone 1 and the boundary between NH zone 1 and 2 over northern Israel should possibly be placed south of Havat Hanania, Israel, at least for the period which is represented by HAN 5B wood growth. This underlines the fact that the boundary between NH zone 1 and 2 needs to be modified with more such Δ^14^C records.

This Havat Hanania Δ^14^C record can be used as a reference for atmospheric Δ^14^C levels in the northern Israel between 1964 and 1968. Earlier, Ehrlich et al.^56^ reported bomb Δ^14^C records from olive and pine latewood collected from Havat Hanania, and they identified the annual nature of olive wood. They further tried to determine the growth season of olive wood by fitting the olive wood’s Δ^14^C record on the available atmospheric and tree ring Δ^14^C records from the northern hemisphere. Use of non-local Δ^14^C records to fit olive wood Δ^14^C records can possibly add some ambiguity to the fitting. However, it is noted that before the present study, no other local continuous atmospheric sub-annual Δ^14^C record existed apart from the Rehovot (Israel) atmospheric record between 1967 and 1968. To clearly understand the growing season of the olive wood, a longer sub-annually resolved atmospheric Δ^14^C record from the local region is required, and the HAN 5B Δ^14^C record provides us with this opportunity.

**Figure 2:**
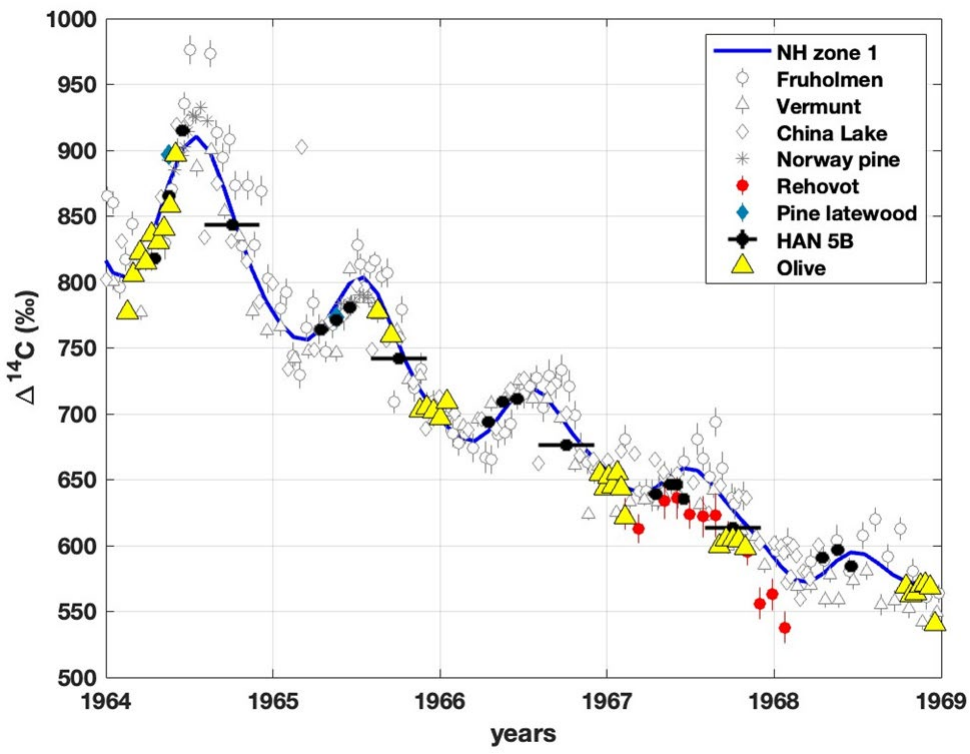
Δ^14^C values from Havat Hanania pine (HAN 5B), Rehovot atmosphere^26^*,* Havat Hanania pine latewood^56^ and olive^56^ along with Fruholmen^26^, Vermunt^27^, China Lake^49–53^ atmospheric Δ^14^C records, Norway pine Δ^14^C records^48^ and monthly NH zone 1 Δ^14^C values^15^*.* Olive data points have been adjusted to match other Δ^14^C records.

Ehrlich et al.^56^ identified sets of olive Δ^14^C values belonging to each year using the olive δ^13^C record. We use the same sets of sub-annual olive Δ^14^C values of each year between 1964 and 1968 and match them with Δ^14^C values of HAN 5B, Rehovot and NH zone 1 (Fig. 2). It will provide a better estimate of the growth period of the olive wood in the region. Figure 2 shows a good match between HAN 5B Δ^14^C record and Rehovot atmospheric Δ^14^C record which demonstrates that HAN 5B record represents well the atmospheric Δ^14^C level of the region. Due to the prominent seasonality in atmospheric Δ^14^C values between 1964 and 1968, the olive Δ^14^C record can be clearly matched to other Δ^14^C records. As olive Δ^14^C values show a clear increasing and decreasing trend in 1964 and 1965, respectively, olive Δ^14^C values can be easily matched with the corresponding trend in the atmospheric Δ^14^C values. The Δ^14^C values matching show that olive wood grew mainly during spring and early summer in the year 1964. However, the next year, in 1965, olive wood grew later, from late summer to winter. Unlike 1964 and 1965, the next three years olive Δ^14^C values show relatively smaller sub-annual variation, and these values are lower than the corresponding year’s HAN 5B Δ^14^C values. The lower Δ^14^C values of olive compared to HAN 5B in 1966, 1967, and 1968 suggest that olive did not grow in the spring or summer period of these years. For the year 1967, when a nearby (Rehovot) atmospheric Δ^14^C record is available, olive Δ^14^C values are matched to that (Rehovot) atmospheric Δ^14^C record. Due to relatively smaller sub-annual Δ^14^C variation in olive Δ^14^C values of 1966, 1967 and 1968, these values can be matched to different atmospheric Δ^14^C values ranging from autumn to winter, and it suggests that olive wood grew sometime between autumn and winter periods in these years. By just looking at records between 1964 and 1966, it is evident that the olive wood growth season shifted from one year (ring) to the other. Understanding the reason for such a shift in olive growth season is beyond the scope of this study, and so it is not discussed here. However, the observation suggests that olive wood can record in some years high ^14^C levels of early summer and also can record relatively low ^14^C levels of winter in other years. When olive wood records low Δ^14^C values of winter, it can possibly reflect offset compared to tree Δ^14^C records from central and northern Europe and North America (growing during the spring–summer period), which constitute major parts of the calibration curve (IntCal). To check for ^14^C offset, annual mean Δ^14^C values of HAN 5B and olive wood were compared with annual mean Δ^14^C values of trees from NH zone 1 (Figure S6), and it is observed that olive Δ^14^C values were clearly lower than NH zone 1 tree values apart from the year 1964. As olive appears to have grown around spring and early summer in 1964, its growth period overlapped with that of central and northern Europe and North American trees, and thus in 1964, its Δ^14^C values were within the observed range of Δ^14^C values of NH zone 1 trees. For the next three years, olive appears to have grown around autumn and winter, and its Δ^14^C values are well below the observed range of Δ^14^C values of central and northern Europe and North American trees. However, pine (HAN 5B) which was growing close to the olive location, always shows Δ^14^C values comparable to central and northern Europe and North American trees. This highlights the possibility of offset in olive wood's annual average Δ^14^C value from the region.

The large seasonal variation in atmospheric bomb Δ^14^C values between 1964 and 1968 exaggerates the offset arising due to the difference in the growing season, and it is more than 25 ‰. For the pre-bomb period, seasonal variation in atmospheric Δ^14^C is inferred to be around 4 ‰^8^, and so the offset arising from the difference in growing season would be much less in the pre-bomb period. An offset of about 2.5 ‰ for plant materials growing in the northern hemisphere but in season opposite to that of central and northern Europe and North American trees has been observed^57,58^. A similar offset can be expected in olive wood ^14^C records from pre-bomb periods, and it can significantly influence calibrated age of events dated using olive wood radiocarbon dates, such as the Minoan eruption event of Santorini^59,60^. The Minoan volcanic event of Santorini is an important time marker in the archaeological records of the eastern Mediterranean region, however, the date of this event is still under scientific discussion. Disagreement between this event's archaeological and radiometric age estimates did not allow the scientific community to reach a consensus about the event date. Various investigation has been conducted to constrain this Minoan volcanic event date, and among them, the one based on radiocarbon dating of an olive branch from Santorini is widely discussed^56,59^. The radiocarbon dating of this olive branch put this event in the seventeenth century BCE, but the archaeological evidence places it in the sixteenth century BCE^59^. Ehrlich et al.^56^ had shown that ^14^C dates of this olive branch can be reconciled with archaeological evidence under some possible scenarios. As seen in this study, the presence of offset in the olive wood ^14^C value can be expected. Such offsets can also influence the calibrated calendar age of the olive branch from Santorini. Applying an offset of 2.5 ‰ calibration model of Santorini olive wood ^14^C dates can clearly increase the probability for younger event dates, mostly in the sixteenth century BCE (Figure S7). Pearson et al.^60^ had also shown that model results with an offset of 13.7 ± 2 ^14^C years increased the probabilities of the sixteenth century BCE date for the Minoan eruption event. However, with current understanding, it is difficult to ascertain if these olive wood ^14^C dates had the offset arising from a shift in the growing season. Nevertheless, this study highlights that offset in olive wood ^14^C dates is possible, and caution while interpreting such dates is warranted.

## Summary

A pine (*Pinus halepensis*) wood sample from Havat Hanania in northern Israel between 1964 and 1968 was analyzed for its ^14^C concentrations. It is observed that the Δ^14^C values of the analyzed pine wood are closer to the atmospheric bomb radiocarbon value of NH zone 1 than NH zone 2 bomb ^14^C values. This observation underlines the requirement to refine the boundary between NH zone 1 and 2 over northern Israel. The pine Δ^14^C record also allowed us to assess the growth period of olive wood from the same region. Δ^14^C values show that the olive wood growth season changed from one year to another. Such growth season shifts in olive wood can result in ^14^C offsets compared to IntCal curve records. So, ^14^C ages estimated from olive woods should be carefully interpreted considering the possibility of ^14^C offset.

## Methods

### Study area and sampling

**Figure 3:**
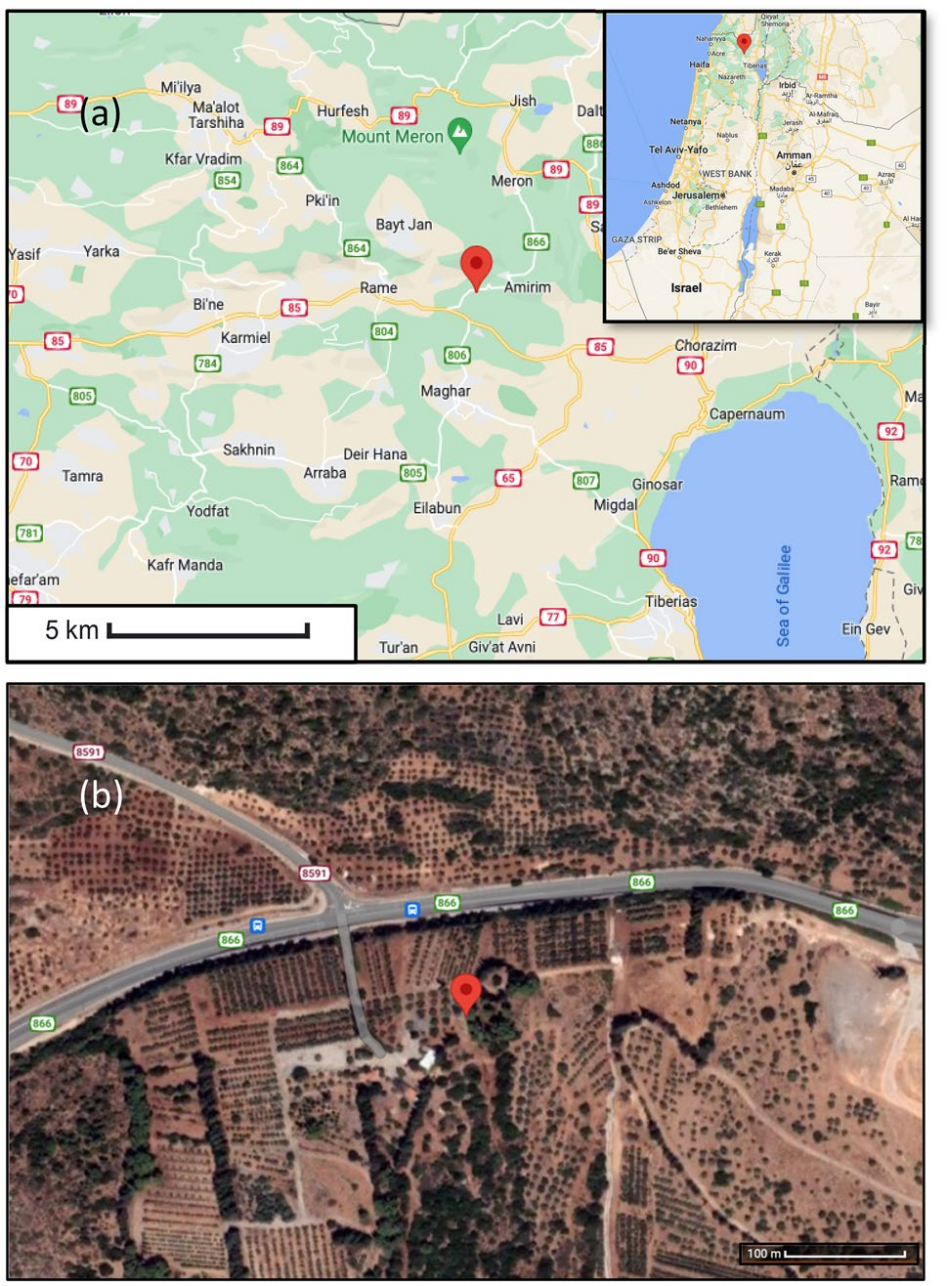
(**a**) Sampling location represented by red map pin in the northern Israel region map (https://www.google.com/maps); (**b**) Satellite image of the sampling location (https://www.google.com/maps).

Pine tree (*Pinus halepensis*) was sampled from Havat Hanania (N32°56.153′, E35°25.296′, 415 m) in northern Israel. Figure 3 shows the sampling location of the pine tree from northern Israel analyzed in the present study. The sampling area was within the property of the Olive Division of the Plants Production and Marketing Board. The sampling was coordinated and approved by the head of the Olive Division and was conducted during the years 2013–2014. The plant collection and use were in accordance with all the relevant guidelines. A cylindrical core was obtained using a 5.15 mm increment borer. The cores were polished using an orbit sander (Makita #BO5041) with five grades of grit (240, 320, 600, 800, 1000). Pine tree rings were visually identified using a binocular microscope (M80, Leica). Pine ring width was measured to the nearest 0.001 mm with the help of a sliding micrometer stage (“TA” measurement system, Velmex Inc.). The Tellervo dendrochronological analysis package^61^ was then used to cross-date the pine ring width data with the master pine ring width record of the region. After cross-dating, rings between 1964 and 1968 were selected for sub-sampling (Figure S1). The sub-sampling was carried out using a WSL-Lab microtome^62^. About 10 mg of wood samples were collected by pooling thin sections cut from a sampling region. Samples were pooled for every 1 mm step, yielding the desired sample size of about 10 mg of pine wood. These pooled samples were processed further for alpha-cellulose extraction.

### Alpha-cellulose extraction

Before processing samples for alpha-cellulose extraction, all glassware required for the process was baked at 450 °C for 1 h to remove organic contaminants. Pooled thin sections of wood (about 10 mg) were placed in borosilicate test tubes (16 × 125 mm).

From these samples, alpha-cellulose was extracted using steps given by Ehrlich et al.^56^ with a modification. We skipped the last acid step of the ABA process done by Ehrlich et al.^56^. This step is for removal any absorbed atmospheric CO_2_ during the previous base step. The last step of holocelluse extraction also includes treatment with acid for absorbed atmospheric CO_2_ removal^56^. Therefore, the last acid step of the ABA process by Ehrlich et al.^56^ was excluded from the present pretreatment process. In present pretreatment, samples were treated with 5 ml of 1N HCl for 1 h, followed by washing with DDW (double distilled water). Then samples were treated with 5 ml of 0.1N NaOH for 1 h, followed by washing with DDW. Then a mix of 2.5 ml of HCl and 2.5 ml of NaClO_2_ was added to each sample and was kept at 70 °C until samples were bleached. After the bleaching step, samples were washed again with DDW. The washed samples were treated for 1 h with 6 ml of 5N NaOH and again washed with DDW. Then samples were treated with 5 ml of 1N HCl at 70 °C for 1 h, followed by a wash with DDW until reaching a neutral pH. The extracted alpha-cellulose was then placed in an oven at 100°C for drying.

### Stable carbon isotope and radiocarbon analysis

About 2–4 mg of alpha-cellulose were weighed and packed in tin foil capsules (Elemental Microanalysis Ltd. 5 × 3.5 mm #D1015). Packed samples were combusted in an Elemental Analyser (Elementar vario ISOTOPE select) linked to an Isotope Ratio Mass Spectrometer (Elementar AMS precision IRMS) and an Automated Graphitization Equipment (Ionplus AGE 3). A fraction of CO_2_ resulting from sample combustion in Elemental Analyser is analyzed in the IRMS and the rest is graphitized over iron powder by AGE 3. For δ^13^C measurements, samples were analyzed along with the NBS SRM-4990C oxalic acid II and IAEA-C3 cellulose^63^ standards, and the precision for these measurements was 0.2 ‰.

For radiocarbon analysis, graphitized samples were pressed into aluminium targets. The targets containing samples and standards are then analyzed in the 500 kV NEC Accelerator Mass Spectrometer at DANGOOR Research Accelerator Mass Spectrometry (D-REAMS) Laboratory in the Weizmann Institute of Science^64^. To check the accuracy of the radiocarbon measurements, VIRI D^65^ and VIRI M^66^ standards were analyzed along with the samples. VIRI D and VIRI M yielded an average value of 2856 ± 18 BP and 73.884 ± 0.072 pMC respectively, which are in very good agreement with the consensus value (VIRI D: 2836 ± 3.3 BP; VIRI M: 73.900 ± 0.0322 pMC)^65,66^ of these standards.

### Supplementary Information

Supplementary Information.

## Data Availability

All data generated or analyzed during this study are included in this published article (and its Supplementary Information files).
