# Supplementary material for: Sub-annual bomb radiocarbon records from trees in northern Israel
Source: Sci Rep. 2023 Nov 1;13:18851. doi: 10.1038/s41598-023-46144-6 (PMC10620229; doi:10.1038/s41598-023-46144-6)
Supplement: Supplementary file 1 — Supplementary Information. [file 41598_2023_46144_MOESM1_ESM.docx]

**Sub-annual bomb radiocarbon records from trees in northern Israel**

Harsh Raj^1^, Yael Ehrlich^1^, Lior Regev^1^, Eugenia Mintz^1^, Elisabetta Boaretto^1^

^1^D-REAMS Radiocarbon Laboratory, Scientific Archaeology Unit, Weizmann Institute of Science, Rehovot, Israel

**Supplementary material**

**Content:**

**Table S1:** δ^13^C and Δ^14^C record of HAN 5B

**Figure S1:** Image of the HAN 5B core used in the present study

**Figure S2:** Plot of Δ^14^C values of earlywood and latewood of HAN 5B

**Figure S3:** Plot of δ^13^C values of earlywood and latewood of HAN 5B

**Figure S4:** Comparison of Δ^14^C values of Havat Hanania pine (HAN 5B) and atmospheric Δ^14^C records from north zone 1 and 2 sites, along with compiled monthly Δ^14^C of these zones

**Figure S5:** Map of wind climatology over the Mediterranean region

**Figure S6:** Comparison of annual average Δ^14^C values of HAN 5B, olive and NH zone 1 tree records

**Figure S7:** Probability distribution of modelled age range for olive wood ^14^C dates

**Table S1:** Radiocarbon and stable carbon isotope values of Pinus halepensis (HAN 5B) samples from Hanania (northern Israel)

| **S. No.** | **Sample ID** | **Wood type** | **Year** | **ẟ^13^C (‰)** | **Δ^14^C (‰)** | **1σ (Δ^14^C)** |
| --- | --- | --- | --- | --- | --- | --- |
| 1 | 11707 | Earlywood | 1964a | -24.5 | 818 | 2 |
| 2 | 11706 | Latewood | 1964b | -23.0 | 866 | 2 |
| 3 | 11705 | Latewood | 1964c | -20.6 | 915 | 3 |
| 4 | 11704 | Earlywood | 1964d | -21.3 | 843 | 3 |
| 5 | 11703 | Earlywood | 1965a | -24.2 | 764 | 2 |
| 6 | 11702 | Latewood | 1965b | -23.2 | 771 | 3 |
| 7 | 11701 | Latewood | 1965c | -20.7 | 780 | 3 |
| 8 | 11700 | Earlywood | 1965d | -21.5 | 742 | 2 |
| 9 | 11699 | Earlywood | 1966a | -24.4 | 693 | 2 |
| 10 | 11698 | Latewood | 1966b | -23.2 | 709 | 2 |
| 11 | 11697 | Latewood | 1966c | -20.2 | 711 | 2 |
| 12 | 11696 | Earlywood | 1966d | -21.5 | 677 | 2 |
| 13 | 11695 | Earlywood | 1967a | -25.2 | 639 | 2 |
| 14 | 11694 | Latewood | 1967b | -23.2 | 647 | 2 |
| 15 | 11693 | Latewood | 1967c | -21.8 | 646 | 2 |
| 16 | 11692 | Latewood | 1967d | -21.6 | 635 | 2 |
| 17 | 11691 | Earlywood | 1967e | -22.5 | 614 | 2 |
| 18 | 11690 | Earlywood | 1968a | -22.6 | 591 | 2 |
| 19 | 11689 | Latewood | 1968b | -23.4 | 597 | 3 |
| 20 | 11688 | Latewood | 1968c | -21.7 | 584 | 2 |


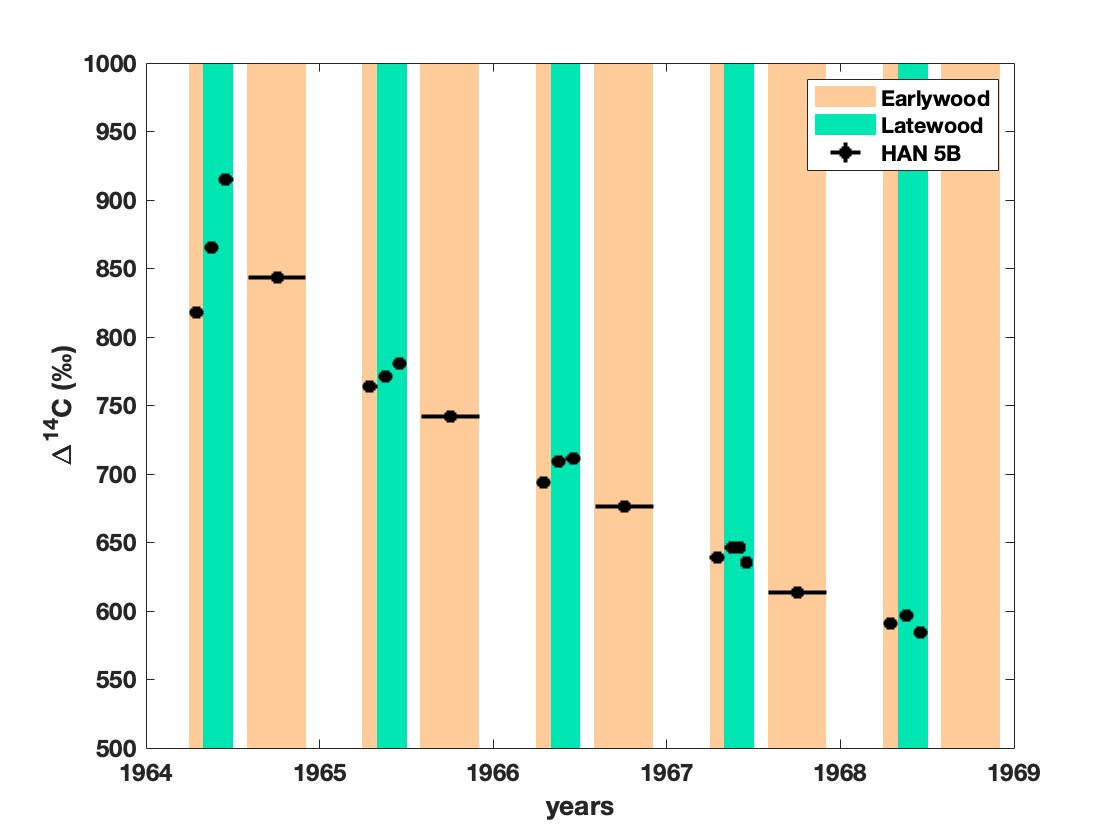
**Figure S2:** Δ^14^C values of Havat Hanania pine (HAN 5B) earlywood and latewood samples between the years 1964 and 1968. The time period of earlywood (light orange area) and latewood (green area) growth of HAN 5B (Pinus halepensis) is based on Liphschitz et al ^42^.


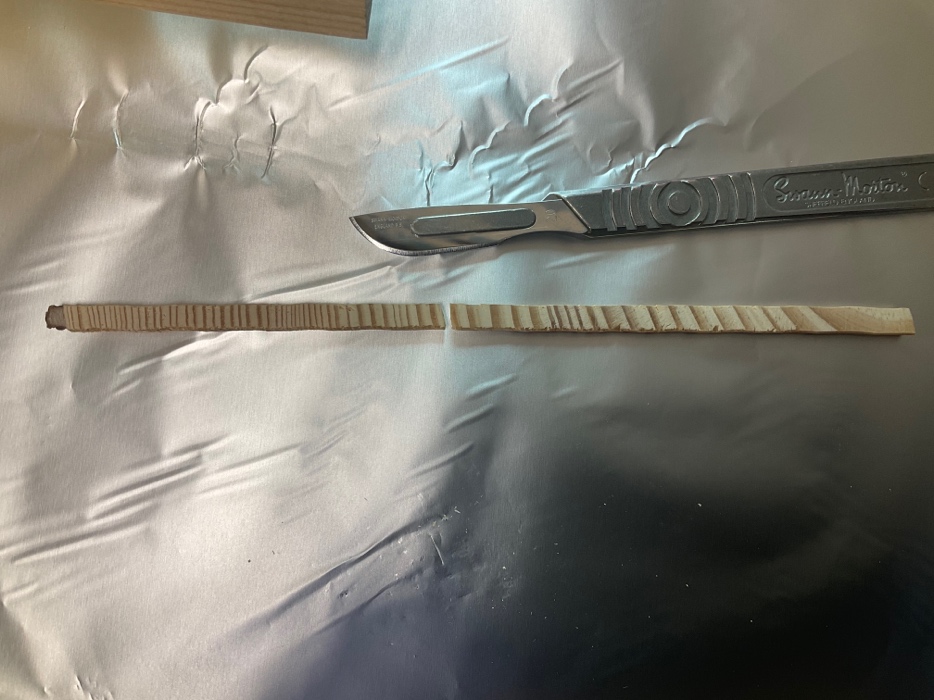

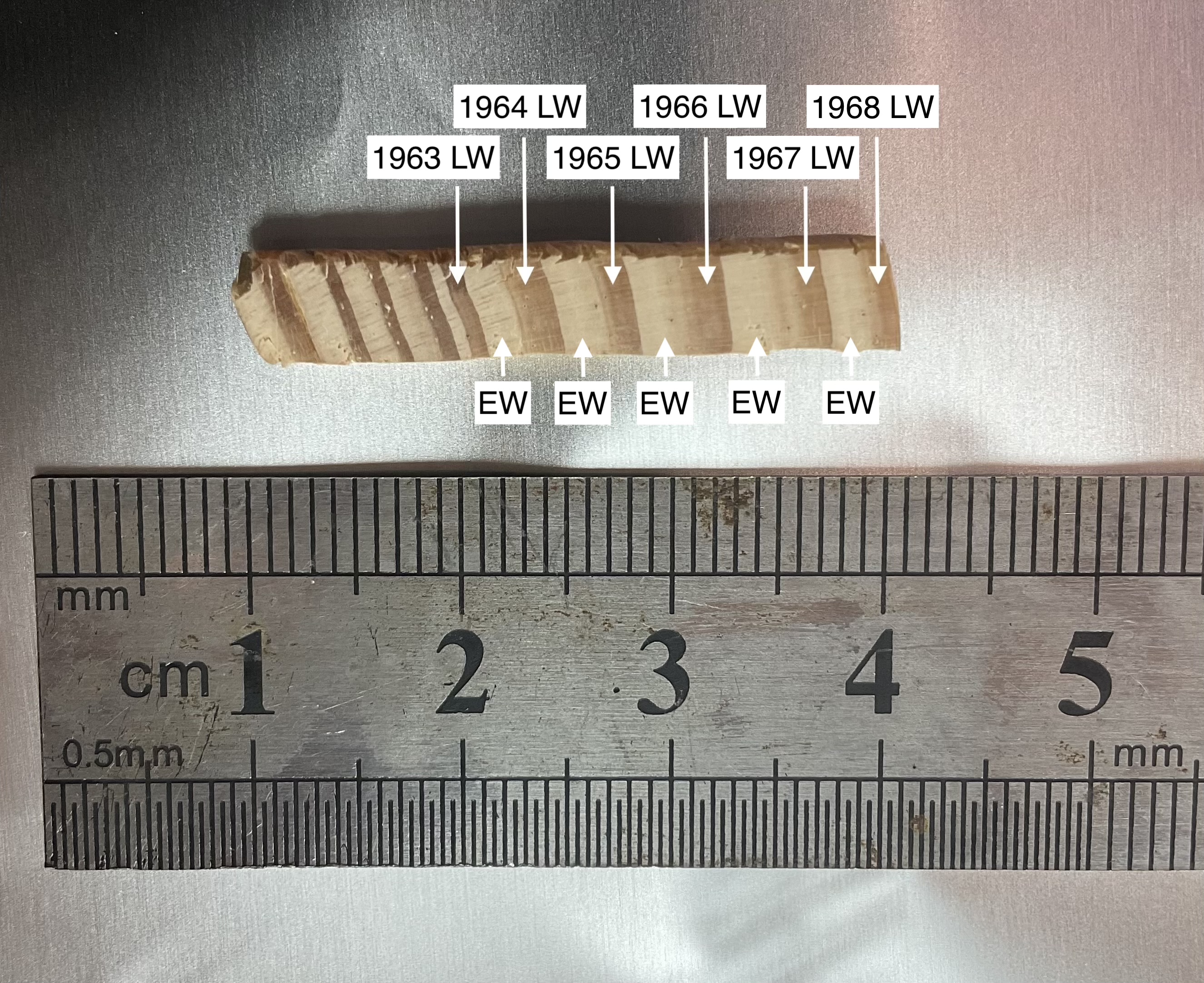


**Figure S1**: Image of the HAN 5B core used in the present study


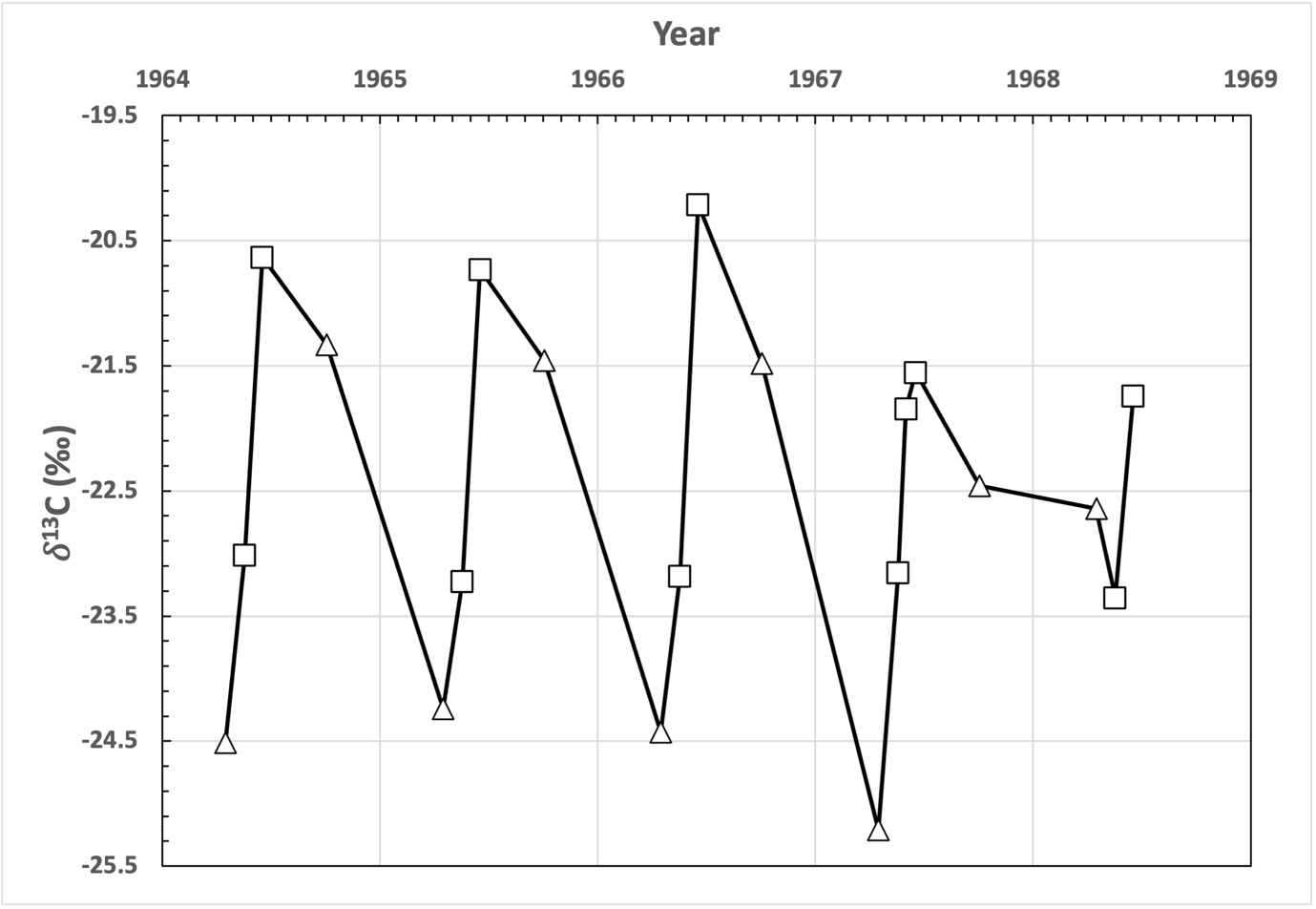


**Figure S3:** δ^13^C values of earlywood (triangles) and latewood (squares) samples from Havat Hanania pine (HAN 5B) between the years 1964 and 1968. δ^13^C values show sub-annual cyclic behaviour.


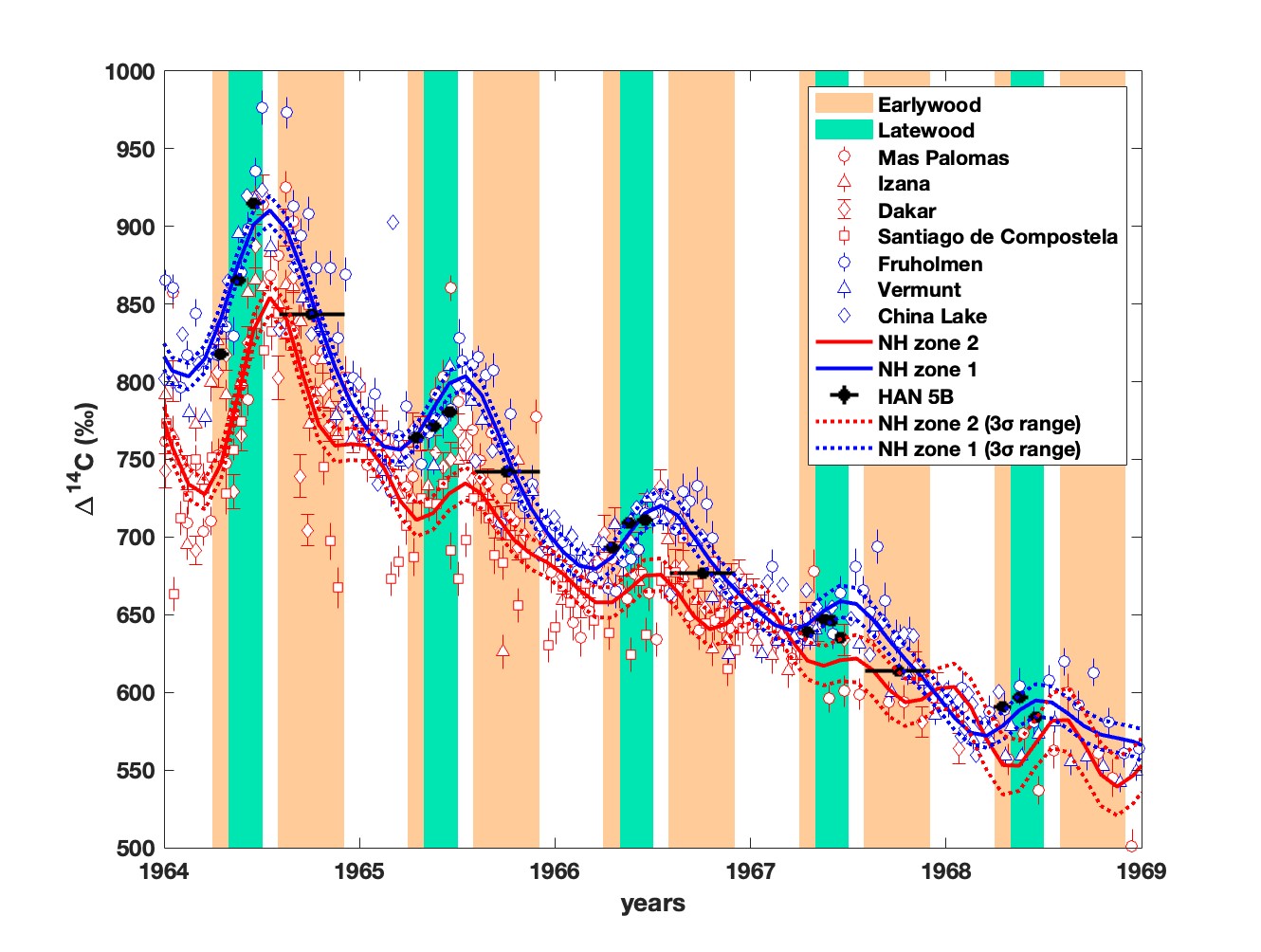


**Figure S4:** Comparison of Δ^14^C values of Havat Hanania pine (HAN 5B) and atmospheric Δ^14^C records from north zone 1 and 2 sites, along with compiled monthly Δ^14^C of these zones (represented by red and blue lines). The dotted lines represent the 3σ range of monthly Δ^14^C of NH zone 1 and 2. The time period of earlywood (light orange area) and latewood (green area) growth of HAN 5B (Pinus halepensis) is based on Liphschitz et al^42^.

**Figure S5:** Monthly average wind climatology (NCEP-NCAR Reanalysis dataset) for month of (a) January and (b) July. Black dot represents the present study location of Havat Hanania in Israel. (Wind map is obtained from https://iridl.ldeo.columbia.edu)


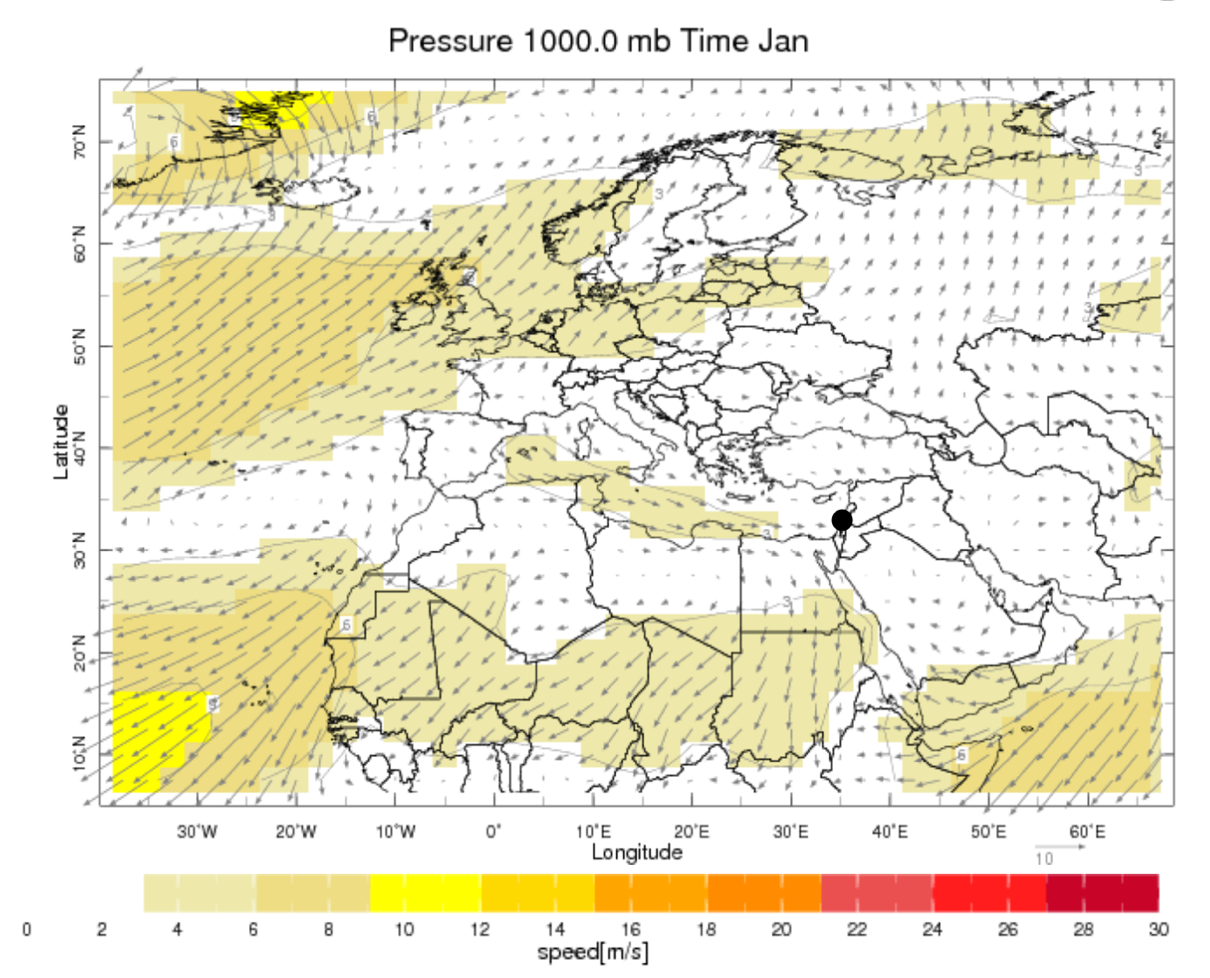

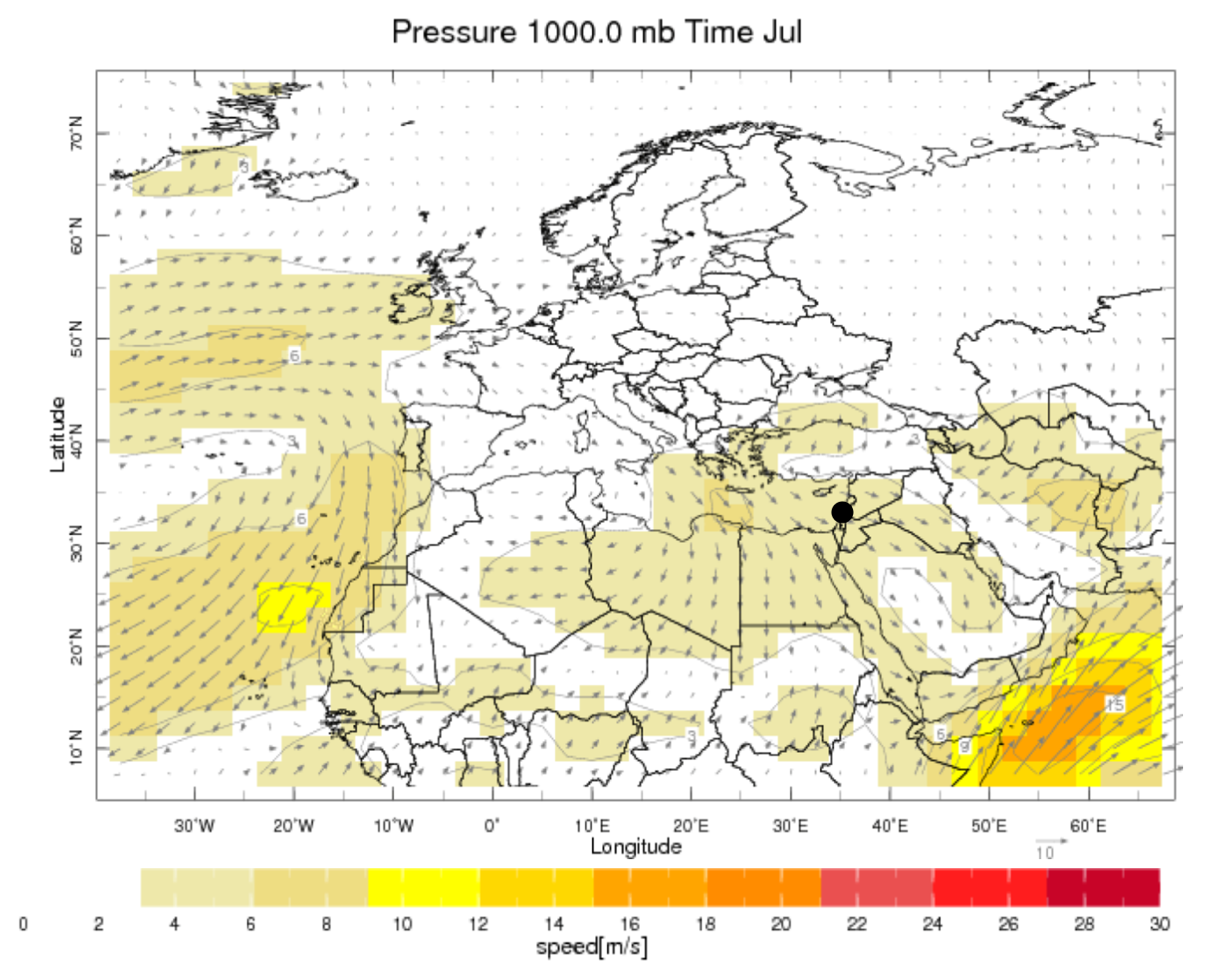


**(a)**

**(b)**


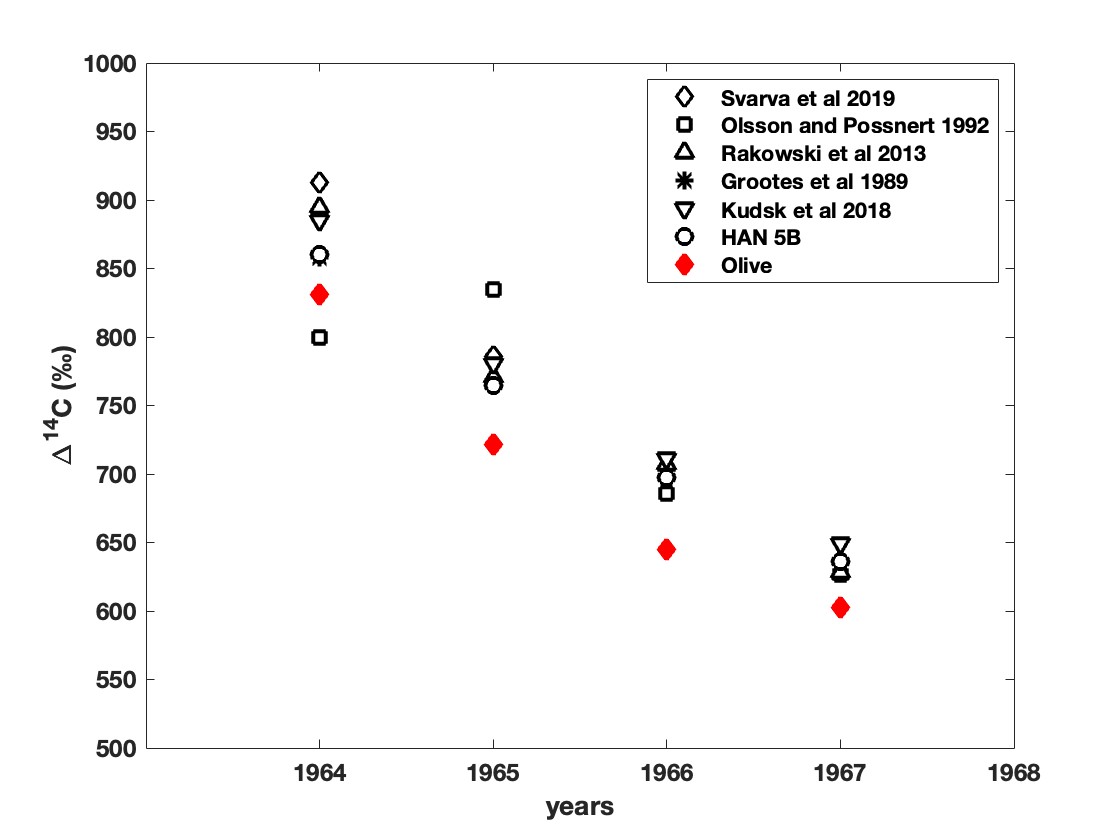


**Figure S6:** Comparison of annual average Δ^14^C values of HAN 5B and olive ^56^ with tree records from NH zone 1 ^48^ ^S1^ ^S2 47^ ^S3^ between 1964 and 1968.


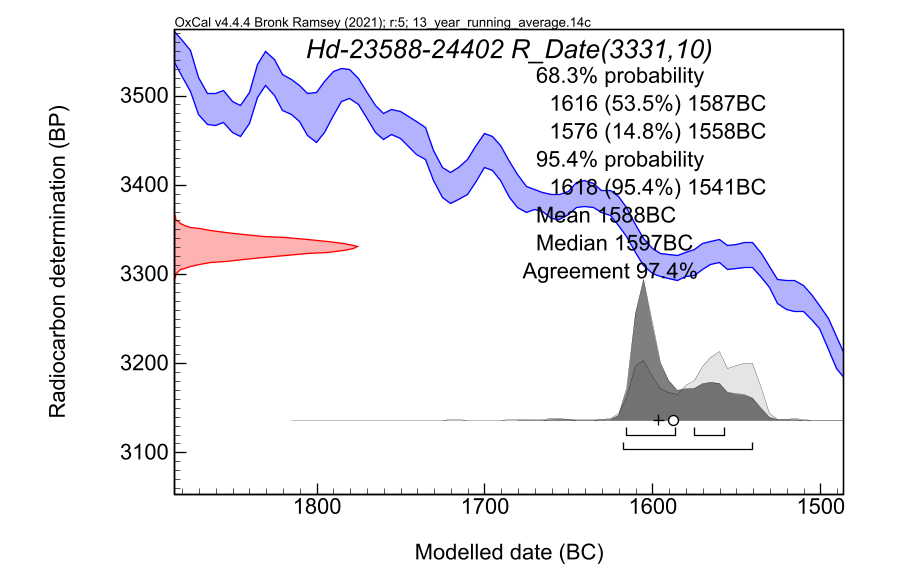

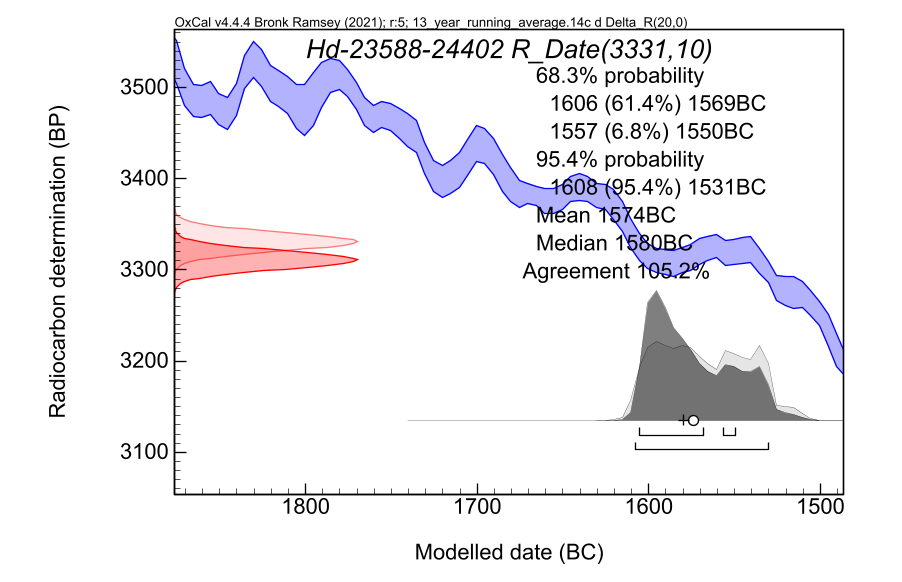


(a)

(b)

**Figure S7:** Probability distribution of modelled age range for olive wood ^14^C dates ^59^ calibrated using moving average calibration curve method ^S4^ in (a) a simple ordered sequence and (b) in a sequence with an offset value of 2.5‰ (20 ^14^C years).

Following is the OxCal model code for the result obtained in Figure S7b:

*Plot( )*

*{*

*Sequence()*

*{*

*Boundary("Start");*

*Curve("", "13_year_running_average");*

*Delta_R("a",20,0);*

*R_Date( "Hd-23599-24426", 3383, 11);*

*Curve("", "24_year_running_average");*

*Delta_R("b",20,0);*

*R_Date( "Hd-23587", 3372, 12);*

*Curve("", "22_year_running_average");*

*Delta_R("c",20,0);*

*R_Date( "Hd-23589", 3349, 12);*

*Curve("", "13_year_running_average");*

*Delta_R("d",20,0);*

*R_Date( "Hd-23588-24402", 3331, 10);*

*Boundary("End");*

*};*

*};*

**Supplementary References**

S1. Olsson, I. U. & Possnert, G. 14 C Activity in Different Sections and Chemical Fractions of Oak Tree Rings, AD 1938–1981. *Radiocarbon* **34**, 757-767 (1992).

S2. Rakowski, A. Z. *et al*. Radiocarbon method in environmental monitoring of CO2 emission. *Nuclear instruments & methods in physics research. Section B, Beam interactions with materials and atoms* **294**, 503-507 (2013).

S3. Kudsk, S. G. K. *et al*. What Is the Carbon Origin of Early-Wood? *Radiocarbon* **60**, 1457-1464 (2018).

S4. Raj, H., Regev, L. & Boaretto, E. Calibration of multiple tree-ring blocks and its implication on the debate of minoan eruption of santorini around 17th–16th century BCE. *Radiocarbon*. doi:10.1017/RDC.2023.35

stylefix
